# Supplementary material for: Construction of a complete set of Neisseria meningitidis mutants and its use for the phenotypic profiling of this human pathogen
Source: Nat Commun. 2020 Nov 2;11:5541. doi: 10.1038/s41467-020-19347-y (PMC7606547; doi:10.1038/s41467-020-19347-y)
Supplement: Supplementary file 1 — Supplementary Information [file 41467_2020_19347_MOESM1_ESM.pdf]

Construction of a complete set of *Neisseria meningitidis* mutants and its use for the phenotypic profiling of this human pathogen

Alastair Muir[1], Ishwori Gurung[1], Ana Cehovin[1], Adelme Bazin[2], David Vallenet[2], Vladimir Pelicic[1,*]

[1]MRC Centre for Molecular Bacteriology and Infection, Imperial College London, London, United Kingdom

[2]LABGeM, Génomique Métabolique, CEA, Genoscope, Institut François Jacob, Université d'Evry, Université Paris-Saclay, CNRS, Evry, France

*Corresponding author
email: v.pelicic@imperial.ac.uk

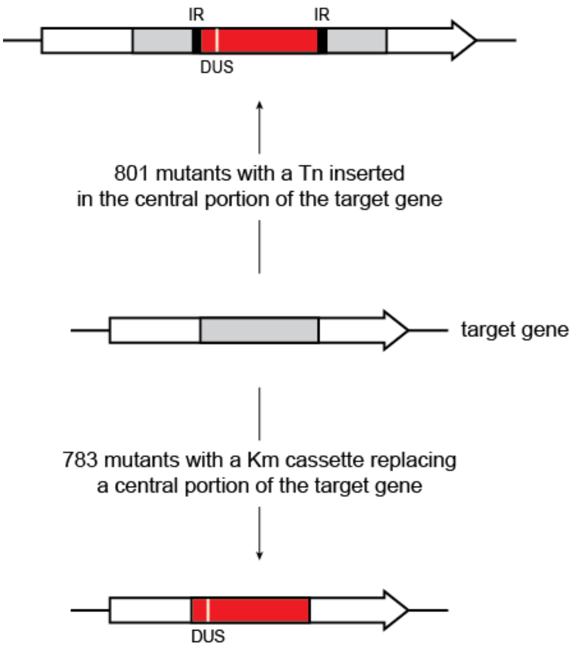

Supplementary Fig. 1. **Cartoon representation of the two types of mutations composing the complete NeMeSys 2.0 library of mutants.** The central portion of the target gene has been highlighted in grey. The cassette is in red and contains a DUS (DNA uptake sequence) necessary for efficient transformation in the meningococcus. In the Tn, the cassette is flanked by IR (inverted repeats).

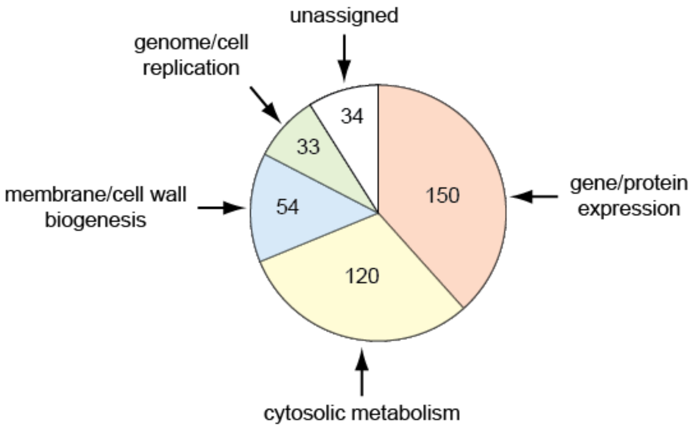

Supplementary Fig. 2. Partition of the meningococcal essential genes into four major functional groups. Gene/protein expression (38.4 %, orange), genome/cell replication (8.4 %, green), cell membrane/wall biogenesis (13.8 %, blue), and cytosolic metabolism (30.7 %, yellow). Only 34 essential genes (8.3 %, white) could not be clearly assigned to one of these four categories. The corresponding datasets are listed in Supplementary Data 8.
