# Supplementary material for: Construction of a complete set of Neisseria meningitidis mutants and its use for the phenotypic profiling of this human pathogen
Source: Nat Commun. 2020 Nov 2;11:5541. doi: 10.1038/s41467-020-19347-y (PMC7606547; doi:10.1038/s41467-020-19347-y)
Supplement: Supplementary file 3 — Description of Additional Supplementary Files [file 41467_2020_19347_MOESM3_ESM.pdf]

Description of Additional Supplementary Files

File Name: Supplementary Data 1
Description: **List of the 1,975 genes in *N. meningitidis* 8013, which were targeted during our systematic mutagenesis, with their essential features.** The 85 genes that were not valid targets are also listed separately.

File Name: Supplementary Data 2
Description: **List of the primers used in this study. a)** List of 6,320 primers that were used for the creation of the complete NeMeSys 2.0 library of mutants. Primers were either used for verifying available Tn mutants (not highlighted), or for sPCR mutagenesis of remaining target genes (highlighted in orange). The 20-mer overhangs in R1 and F2, which are complementary to the primers used to amplify the kanamycin resistance cassette, are in lower case. **b**) List of other primers. Overhangs are in lower case, with restriction sites underlined.

File Name: Supplementary Data 3
Description: **List of the 1,589 meningococcal mutants constituting the complete NeMeSys2.0 library of mutants.** The 801 Tn mutants are not highlighted, while the 788 mutants constructed by sPCR mutagenesis are highlighted in orange.

File Name: Supplementary Data 4
Description: **List of the 391 essential meningococcal genes, which could not be mutagenised.**

File Name: Supplementary Data 5
Description: **Partition of meningococcal genes into persistent (present in almost all meningococci, not highlighted), shell (present at intermediate frequencies, highlighted in gold), or cloud (present at low frequency, highlighted in orange)**

genomes. Partitioning was done using the PPanGGOLiN software on 108 complete *N. meningitidis* genomes, publicly available in RefSeq. The partitioning of the 391 essential genes is also listed separately.

File Name: Supplementary Data 6
Description: **Essentiality conservation of meningococcal essential genes in three other bacteria in which complete libraries of mutants have been constructed.** Two are Gram-negative Proteobacteria (*E. coli* K-12 and *A. baylyi* ADP1), while one is a Gram-positive Firmicute (*S. sanguinis* SK36).

File Name: Supplementary Data 7
Description: **Conservation of meningococcal essential genes in JCVI-syn3.0, a synthetic *M. mycoides* bacterium engineered with a minimal genome.**

File Name: Supplementary Data 8
Description: **Partition of meningococcal essential genes into four major functional groups.** 1) gene/protein expression (150 genes), 2) genome/cell replication (33 genes), 3) cell membrane/wall biogenesis (54 genes), and 4) cytosolic metabolism (120 genes). Only 34 essential genes could not be assigned to one of these functional categories.

File Name: Supplementary Data 9
Description: **List of the 32 RGP in strain 8013, with the genes they encompass.** RGP were identified using the panRGP module of PPanGGOLiN. Essential genes in these RGP are highlighted in orange.

File Name: Supplementary Data 10

Description: **Results of a dual phenotypic screen, using the NeMeSys 2.0 complete library of mutants, for mutants affected for aggregation and/or twitching motility in liquid culture.** The 20 mutants impaired for the formation of aggregates and/or twitching motility are highlighted in orange. The corresponding genes with their essential features are also listed separately.
