# Supplementary material for: Construction of a complete set of Neisseria meningitidis mutants and its use for the phenotypic profiling of this human pathogen
Source: Nat Commun. 2020 Nov 2;11:5541. doi: 10.1038/s41467-020-19347-y (PMC7606547; doi:10.1038/s41467-020-19347-y)
Supplement: Supplementary file 14 — Reporting Summary [file 41467_2020_19347_MOESM14_ESM.pdf]

nature research

Corresponding author(s): Vladimir Pelicic

Last updated by author(s): Sep 25, 2020

Reporting Summary

Nature Research wishes to improve the reproducibility of the work that we publish. This form provides structure for consistency and transparency in reporting. For further information on Nature Research policies, see our Editorial Policies and the Editorial Policy Checklist.

Statistics

For all statistical analyses, confirm that the following items are present in the figure legend, table legend, main text, or Methods section.

n/a	Confirmed	
☐	☒	The exact sample size (n) for each experimental group/condition, given as a discrete number and unit of measurement
☐	☒	A statement on whether measurements were taken from distinct samples or whether the same sample was measured repeatedly
☐	☒	The statistical test(s) used AND whether they are one- or two-sided<br>*Only common tests should be described solely by name; describe more complex techniques in the Methods section.*
☒	☐	A description of all covariates tested
☒	☐	A description of any assumptions or corrections, such as tests of normality and adjustment for multiple comparisons
☐	☒	A full description of the statistical parameters including central tendency (e.g. means) or other basic estimates (e.g. regression coefficient) AND variation (e.g. standard deviation) or associated estimates of uncertainty (e.g. confidence intervals)
☐	☒	For null hypothesis testing, the test statistic (e.g. F, t, r) with confidence intervals, effect sizes, degrees of freedom and P value noted<br>*Give P values as exact values whenever suitable.*
☒	☐	For Bayesian analysis, information on the choice of priors and Markov chain Monte Carlo settings
☒	☐	For hierarchical and complex designs, identification of the appropriate level for tests and full reporting of outcomes
☒	☐	Estimates of effect sizes (e.g. Cohen's d, Pearson's r), indicating how they were calculated

Our web collection on statistics for biologists contains articles on many of the points above.

Software and code

Policy information about availability of computer code

Data collection	Whole-genome sequencing (WGS) was performed by MicrobesNG, using a HiSeq 2500 (Illumina) sequencer. The reads were trimmed using Trimmomatic (version 0.39) and the quality was assessed using Samtools (version 0.1.19), BedTools (version 2.29.2) and bwa-mem (version 0.7.17).
Data analysis	Primer3 was used for primer design either in batch (version 1.1.4) or manually (version 4.1.0). Statistical analyses were performed using GraphPad Prism (version 8.4.3). WGS analysis was performed using QiagenCLC Genomics Workbench software (version 20.0.4). Gene partitioning in persistent, shell and cloud genomes was done using PPanGGOLiN (version 1.1.85). Regions of genome plasticity were identified using the panRGP module in PPanGGOLiN (version 1.1.85). Preliminary genome annotations were viewed in Artemis (version 17.0.1). DNA Strider (version 3.5 z1) was used to perform and view pairwise sequence alignments of genomic regions of interest.

For manuscripts utilizing custom algorithms or software that are central to the research but not yet described in published literature, software must be made available to editors and reviewers. We strongly encourage code deposition in a community repository (e.g. GitHub). See the Nature Research guidelines for submitting code & software for further information.

Data

Policy information about availability of data

All manuscripts must include a data availability statement. This statement should provide the following information, where applicable:
- Accession codes, unique identifiers, or web links for publicly available datasets
- A list of figures that have associated raw data
- A description of any restrictions on data availability

Whole genome sequencing data that support the findings in this study have been deposited in the European Nucleotide Archive under access number PRJEB39197 (https://www.ebi.ac.uk/ena/browser/view/PRJEB39197). All the datasets generated during this study are either included in this paper and its Supplementary

Information files, or available in MicroScope (http://mage.genoscope.cns.fr/microscope/mage/viewer.php?O_id=99). Source data are provided with this paper. The RefSeq database (http://www.ncbi.nlm.nih.gov/refseq/) was used to access annotations of publicly available genomes of N. meningitidis. Lists of essential genes in other bacteria were obtained from the DEG repository (http://tubic.tju.edu.cn/deg/).

Field-specific reporting

Please select the one below that is the best fit for your research. If you are not sure, read the appropriate sections before making your selection.

- [x] Life sciences
- [ ] Behavioural & social sciences
- [ ] Ecological, evolutionary & environmental sciences

For a reference copy of the document with all sections, see nature.com/documents/nr-reporting-summary-flat.pdf

Life sciences study design

All studies must disclose on these points even when the disclosure is negative.

Sample size	Number of replicate experiments were not predetermined on statistical methods, but were chosen according to common practice in molecular biology. In brief, at least three independent experiments were performed, with exact replicate numbers given in figure legends. Statistical analysis was limited to quantitative assays, the results of which were submitted to multiple comparison tests (one-way ANOVA, followed by Dunnett's) to estimate statistical significance of the observed differences.
Data exclusions	No data was excluded from the analyses.
Replication	In general, experiments were repeated at least three times to ensure reproducibility including growth assays, pilus preps, SDS-PAGE analysis (both Comassie and immunoblots), aggregation assays, and competence assays. For example, to minimise false positive identification of essential genes, each transformation that yielded no mutants was repeated at least three times. Only if all three repeats failed to produce mutants was the the gene declared essential. For the quantitative assays (ELISA and competence), experiments were performed between four and five times (the exact number of repeats is indicated in the figure legends) to ensure statistical significance in multiple comparison tests.
Randomization	Randomization was not necessary as no allocation of samples into experimental groups was required.
Blinding	During the mutagenesis experiments, the investigators were blinded to the identity of the gene that was targeted, which was identified only by an NMV_ identifier. This was done to ensure that no prior assumption would be made on putative essentiality of the gene, avoiding bias. This was also the case during the phenotypic screening for mutants affected for T4P functions.

Reporting for specific materials, systems and methods

We require information from authors about some types of materials, experimental systems and methods used in many studies. Here, indicate whether each material, system or method listed is relevant to your study. If you are not sure if a list item applies to your research, read the appropriate section before selecting a response.

Materials & experimental systems

n/a	Involved in the study	
[ ]	[x]	Antibodies
[x]	[ ]	Eukaryotic cell lines
[x]	[ ]	Palaeontology and archaeology
[x]	[ ]	Animals and other organisms
[x]	[ ]	Human research participants
[x]	[ ]	Clinical data
[x]	[ ]	Dual use research of concern

Methods

n/a	Involved in the study	
[x]	[ ]	ChIP-seq
[x]	[ ]	Flow cytometry
[x]	[ ]	MRI-based neuroimaging

Antibodies

Antibodies used	The primary antibodies – 20D9 mouse monoclonal antibody that is specific for the type IV pili in strain 8013, and anti-PilE rabbit serum raised against the major subunit of type IV pili in strain 8013 – have been obtained from Prof. Xavier Nassif (Institut Necker Enfants Malades, France) who produced them. The secondary antibodies were purchased from GE Healthcare: Amersham ECL anti-mouse IgG HRP-linked whole antibody (NXA931, lot363941) and Amersham ECL anti-rabbit IgG HRP-linked whole antibody (NA934, lot17041905).
Validation	The 20D9 mouse monoclonal antibody has been described and validated in PMID: 9353073. The anti-PilE rabbit serum has been described and validated in PMID: 15103324.
